# Unmet healthcare needs and health inequalities in people with spinal cord injury: a direct regression inequality decomposition

**DOI:** 10.1186/s12939-023-01848-z

**Published:** 2023-03-30

**Authors:** Ana Oña, Kyriakides Athanasios, Piotr Tederko, Reuben Escorpizo, Mohit Arora, Christian Sturm, Shujuan Yang, Diana Pacheco Barzallo

**Affiliations:** 1 Swiss Paraplegic Research, Guido A. Zäch Institute, Nottwil, Switzerland; 2 Department of Health Sciences and Medicine, University of Lucerne, Lucerne, Switzerland; 3 Spinal Cord Rehabilitation Unit, Medical University of Patras, Patras, Greece; 4 Department of Rehabilitation, Medical University of Warsaw, Warsaw, Poland; 5 The University of Vermont, Burlington, USA; 6 John Walsh Centre for Rehabilitation Research, The Kolling Institute, Royal North Shore Hospital, Northern Sydney Local Health District, Sydney, Australia; 7 Faculty of Medicine and Health, Sydney Medical School – Northern, The University of Sydney, Sydney, Australia; 8 Department of Rehabilitation Medicine, Hannover Medical School, Hanover, Germany; 9 China School of Public Health and West China Fourth Hospital, Sichuan University, Chengdu, China; 10 International Institute of Spatial Lifecourse Epidemiology (ISLE), Beijing, China; 11 Center for Rehabilitation in Global Health Systems, WHO Collaborating Center, Lucerne, Switzerland

**Keywords:** Health disparities, Bivariate inequality, Socioeconomic health inequality, Comorbidity index, Spinal cord injury

## Abstract

**Background:**

Inequality in health is a prevalent and growing concern among countries where people with disabilities are disproportionately affected. Unmet healthcare needs explain a large part of the observed inequalities between and within countries; however, there are other causes, many non-modifiable, that also play a role.

**Aim:**

This article explores the difference in health across income levels in populations with spinal cord injury (SCI). SCI is of special interest in the study of health systems, as it is an irreversible, long-term health condition that combines a high level of impairment with subsequent comorbidities.

**Methods:**

We estimated the importance of modifiable and non-modifiable factors that explain health inequalities through a direct regression approach. We used two health outcomes: years living with the injury and a comorbidity index. Data come from the International Spinal Cord Injury Survey (InSCI), which has individual data on people with SCI in 22 countries around the world. Due to the heterogeneity of the data, the results were estimated country by country.

**Results:**

On average, the results exhibit a prevalence of pro-rich inequalities, i.e., better health outcomes are more likely observed among high-income groups. For the years living with the injury, the inequality is mostly explained by non-modifiable factors, like the age at the time of the injury. In contrast, for the comorbidity index, inequality is mostly explained by unmet healthcare needs and the cause of the injury, which are modifiable factors.

**Conclusions:**

A significant portion of health inequalities is explained by modifiable factors like unmet healthcare needs or the type of accident. This result is prevalent in low, middle, and high-income countries, with pervasive effects for vulnerable populations like people with SCI, who, at the same time are highly dependent on the health system. To reduce inequity, it is important not only to address problems from public health but from inequalities of opportunities, risks, and income in the population.

**Highlights:**

• Better health status is evident among high-income groups, which is reflected in pro-rich inequalities.

• Age at the time of the injury is the most important factor to explain inequalities in years living with the injury.

• Unmet health care needs are the most important factor to explain inequalities in comorbidities.

• The inequality in health varies by country dependent upon socioeconomic factors.

**Supplementary Information:**

The online version contains supplementary material available at 10.1186/s12939-023-01848-z.

## Introduction

Despite the remarkable progress in health indicators worldwide, inequalities persist and in many cases are increasing between and within countries [1], a reality that became more evident after the COVID-19 pandemic [2]. Inequality affects the health and well-being of everyone, but with more pervasive effects on vulnerable groups, such as people facing disabilities. In general, people with disabilities have a reduced health status, but also experience several disadvantages compared to the general population [3–5]. In fact, when a family member faces a disability, the probability of poverty increases several times, especially in countries with weak social support systems [6–8]. As the number of people facing disability is expected to steadily increase worldwide [5], policies aiming at identifying the source of health inequalities become more relevant.

Existing evidence suggests that a big part of inequalities in health are modifiable [4, 9] but difficult to address as their causes start early in people’s lives [10]. Many causes go beyond the availability and provision of healthcare and include the social conditions into which people are born, like their financial situation, or their working conditions [11, 12]. Towards health equity and to guarantee an appropriate response from the health system to an increasing number of people, the sources of such inequality must first be identified [13, 14] and quantified to better inform policymakers. Although studies on inequalities have increased, these have focused on the general population or disabilities globally. People with disabilities are a very diverse group and it is required specific studies to better understand their needs and realities, which differ from one disability to another [5].

This study aims to understand the composition of the socioeconomic inequality of health in people with spinal cord injury (SCI). We estimated to what extent unmet healthcare needs explain health inequalities by computing and decomposing a *concentration index*. In general, unmet healthcare needs are largely explained by failures and limitations in the provision of healthcare services [3, 15, 16], and, to a lesser extent, by external factors, such as the lack of transportation, inadequate information, or other cultural factors [15, 17]. However, not all factors contribute in the same way to health inequalities. This paper applies the direct regression approach proposed by Kessels and Erreygers (2019) to identify the importance of different factors in observed health inequality in people with SCI. The analysis uses data from the International Spinal Cord Injury Survey (InSCI), which is the first survey with comparable data across 22 countries [18–20].

We focus on SCI as it is a long-term health condition that can generate higher levels of disability as it combines physical impairment (paraplegia/tetraplegia) with a series of comorbidities [21]. Thus, persons with SCI are highly dependent on the efficiency of the health system, as they require frequent access to a variety of services across the continuum of care, including paramedics, general physicians, specialized care, and community care [22, 23]. This characteristic makes SCI one of the groups with the highest needs and costs in the health system, even when its incidence is quite low [21]. In fact, the mortality risk shows high variations, while in developed countries persons with SCI are living, on average, more than 20 years after the injury [21, 24], in developing countries persons with SCI have a much shorter life span as they die just after the injury, or by preventable secondary health conditions [21], which highlights the relevance of the health system for this group.

## Methods

This is a cohort study that uses a cross-sectional survey. To conduct our analysis, we followed three steps. First, we calculated a concentration index of health according to the income distribution in each country. For the analysis, we focused on two health outcomes that are relevant for people with SCI: 1) the number of years living with the injury, and 2) a comorbidity index. While the first outcome gives a general overview of the survival situation of persons with SCI in each country [24], the comorbidity index serves as a proxy for mortality [25–27]. Second, we followed related literature to identify the factors that explain health inequalities in related groups. Identifying such factors is challenging in theoretical, methodological, practical, and moral terms, as not all the factors that generate differences in health also generate inequalities [4, 11], and not all of these factors are available in the data set. Finally, we calculated the relative weight of each factor in health–income inequality through a direct regression approach to decompose socioeconomic inequality of health.

### Data

Data come from the International SCI community survey (InSCI) which is the first comparable data about the lived experience of people with SCI in 22 countries around the world [18]. Due to the lack of reliable information on the prevalence and incidence of SCI worldwide, this survey is an important input to better understand the living situation of people with SCI across countries. Data were collected between 2017 and 2018 and included 12,590 participants [19]. Eligibility criteria included participants older than age 18 with traumatic or non-traumatic SCI. The sample excluded persons with spinal cord damage due to congenital etiologies and those receiving their first rehabilitation on the date of the survey [20].

The questionnaire used the International Classification of Functioning, Disability, and Health (ICF) framework for capturing the challenges and barriers that SCI populations face [18, 20]. The survey comprised 11 modules that included personal information, lesion characteristics, energy and feelings, health problems, activity and participation, independence in activities of daily living, work, environmental factors, healthcare services, personal factors, quality of life, and general health.

The official channels to invite countries to participate in InSCI included the International Society of Physical Rehabilitation and Medicine (ISPRM) and the International Spinal Cord Society (ISCoS). The recruitment and data collection were undertaken by each country; however, how representative these samples were is unknown due to the lack of national registers. In fact, to date, the estimated prevalence of SCI has high variations in developed and developing countries [21]. For this reason, the InSCI study center computed the minimum number of participants per country in at least 200 to allow for comparability between items and countries [18, 20]. Among the 22 countries, Australia, China, Germany, Netherlands, Norway, Poland, South Africa, and Switzerland had a random sample. Brazil, France, Indonesia, Italy, Greece, Japan, Lithuania, Malaysia, Morocco, Romania, South Korea, Spain, Thailand, and the USA, had a convenience sample [19]. Depending on the means available in each country, data were collected using paper–pencil or online questionnaires, and telephone or personal interviews [20].

### Health outcomes

We analyzed two health outcomes to estimate the inequality index that are pertinent to persons with SCI: years living with the injury and a comorbidity index. The first outcome, years living with the injury, provides a general picture of the survival years after the injury across countries [24]. It was computed using the year of the survey minus the year of the injury. The second outcome, a comorbidity index, provides information about the health status of the participants. It was computed using the 15 reported secondary health conditions and weighted by their severity and their correlation with mortality [24].

The comorbidity index ranged from zero (no comorbidities) to 90 (high number of comorbidities); however, in our sample, the maximum observed value reached 54. For methodological and interpretation purposes, the index was re-scaled country by country, so fewer comorbidities took the value of 100 (best health status) and more comorbidities (worse health status) took the value of zero. In the appendix, we detailed how the comorbidity index was built (Appendix A1).

### Socio-economic status

There are different domains to proxy the socioeconomic status of a person, such as the type of occupation, family income, education level, among others [28]. For this paper, due to the different educational and vocational systems across countries, we used equivalized income to approach the socioeconomic level of the participants. Nevertheless, income has been shown to be a good proxy for wellbeing [29]. To compute the equivalized income, we used the reported household income by the participants by country. Household income was collected at levels, in total 10 levels per country defined by the responsible research teams. However, this measure as reported did not allow for comparisons among countries because it did not take into account the income distribution of each country. Therefore, we computed a standardized measure of income that allow us to identify how rich or poor each participant was in their own country, and with respect to the other countries in the sample.

For this process, we first transformed the reported income to dollars 2018 in constant terms. Second, we calculated the net income (income after taxes) for each income level and country to get the disposable income. We implemented random draws (10,000) between the income levels and took the average value for each participant. To get the equivalent income per capita, we adjusted this value by the household size of each participant. To have the income standardized information for each country we used data from the Luxemburg Income Study database (LIS) [30], the World Inequality Database (WID) [31], the European Commission (Eurostat) database [32], and national surveys [24], data that allowed us to estimate the income according to the income distribution of their country.

### Unmet healthcare needs

The item that inquired participants about their unmet healthcare needs in the survey came from two international instruments: The Model Disability Survey I6009 and the World Health Survey 2001–2 (reduced version) [20]. The item asked participants the following:

- *In the last 12 months, have you needed healthcare but did not get it?* Answers: *Yes, No*.

Participants that answered yes were also asked about the reasons for unmet healthcare needs. The multiple options include: *healthcare costs, unavailable service, transportation (cost and availability), badly treated, work-related, inadequate provider, lack of information, and other reasons as they thought that were not sick enough* [19, 20].

### Statistical analysis

To define which factors determine health inequality in people with SCI, we followed two steps for the two analyzed health outcomes. First, we measured the inequality of the distribution by one- and two-dimension concentration indices: Gini index and Erreygers [33]. Second, we implemented a direct regression approach to compute the importance of each factor in the inequality of health outcomes [34].

### Concentration indices

In general, concentration indices are used to measure inequality in one variable of interest due to the distribution of another variable in a given population [35]. For the one-dimension index, the Gini index and the Lorenz curve provide an inequality measure of income or wealth within a group [36]. This analysis is useful to give a general overview of the distribution of one variable.

For the two-dimensions index, the variables measuring health and socioeconomic status need a clearer definition. In this study, the health variables are two, the years living with SCI and the comorbidity index; the socioeconomic status is measured by the estimated equivalized income. While the income variable is standard across countries, health does not have a “natural unit.” Different measures can be used and be as good (or as bad) as any other. For example, health could be measured with a scale from zero to 1, or 1 to 50, or bounded by the values of the sample [35]. As the two health outcomes have a finite upper limit, we implemented the Erreygers concentration index [35]. For a population of $n$ individuals $\left(i=\mathrm{1,2},\dots ,n\right),$ the Erreygers concentration index is defined as follows:

$$E\left(h|y\right)=\frac{1}{n}{\sum }_{i=1}^{n}\left\{\frac{4{h}_{i}}{{(h}^{max}-{h}^{min})}(2{R}_{i}-1)\right\}$$

$$E\left(h|y\right)$$ reveals the concentration index of health outcomes $$(h)$$ by socioeconomic variable, in this case, income ($$y)$$, depending on the fractional (income) rank $$R.$$ This estimation differentiates between good health and high-income vs low health and low-income [37]. If the coefficient is zero, the health outcome does not vary with income rank. If the coefficient is negative, the concentration of health is higher in the poor group than in the non-poor groups (pro-poor). The opposite situation (positive coefficient), reflects a pro-rich situation—more concentration of the health outcome in the rich group [33]. For the first outcome, years living with the injury $${h}^{max}$$ was 85 and $${h}^{min}$$ 0.5, while for the second outcome, the comorbidity index $${h}^{max}$$ was 100 (defined by the transformation) and $${h}^{min}$$ 0.

### Decomposition of the inequality

To identify the importance of each factor in the observed inequality, we decomposed the concentration index. However, many factors and their interrelations determine individual and population health including genetics or behavior, as well as healthcare provision or environment [38], and not everything can be identified. Differences in health that are unnecessary or modifiable generate inequalities that are considered “unfair.” Ideally, we would like to identify all the factors that explain unfair health inequality. However, achieving this goal is challenging, as it requires a clear identification of the “unfair” and “fair” factors [39] in theoretical terms (philosophical, ethics) and in practice (econometric estimations) [12, 13, 40, 41].

This paper uses the direct regression approach proposed by Kessels and Erreygers (2019) to decompose socioeconomic inequality in the analyzed health outcomes. To do so, it was important to first define the factors that could be correlated with income and health indicators. The selection of such factors followed two approaches: One, literature that identifies the most common variables that determine differences in health in people with SCI; two, the available information in the survey. For the first health outcome, years living with the injury, the included factors ${(X}_{j})$ were the age at the time of the injury, gender, type of injury (paraplegia or tetraplegia), degree of the injury (incomplete or complete), unmet healthcare needs, and the traumatic causes of the injury (work accidents, traffic accidents, falls, and other traumatic causes). For the second outcome, the comorbidity index, the included factors were age, years living with the injury, gender, type and degree of injury, unmet healthcare needs, and the cause of the injury.

We assumed that both variables, ${y}_{i}$ (income) and ${h}_{i}$ (health outcome), were ratio-scale variables with well-defined lower bounds greater than or equal to 0 [34]. The limits for years living with injury ranged from 0.5 to 89 years (defined by the sample); for the comorbidity index, the bounds ranged from 0–100 (defined by the construction of the variable). In the case of income, this variable was considered an unbounded variable and changed according to the country’s characteristics.

The direct regression approach uses a bivariate inequality ($${d}_{i}$$), combining the socioeconomic status, income ($${y}_{i})$$, and health $$\left({h}_{i}\right) .$$ This measure can be defined as follows^1^:

$${d}_{i}= {\widetilde{y}}_{i}{h}_{i}-{\mu }_{h}$$

The socioeconomic status serves as the weighing variable for the health indicator [34]. The relative income of an individual ${\widetilde{y}}_{i}$ is defined as the ratio of their income to the average income ${\mu }_{y}$ in the sample, by country; ${\mu }_{h}$ is the average health. ${d}_{i}$ can be interpreted as the performance of each individual in terms of the “reference outcome,” which reflects the average situation of the income–health domain. The reference outcome could be seen as $\left({y}_{i}, {h}_{i}\right)=({\mu }_{y},{\mu }_{h})$. This value is positive if the individual performs better than the average individual in the sample on both income and health variables. Otherwise, the value is negative. If we assume that ${d}_{i}$ is explained by the factors in ${X}_{j}$, the linear regression equation is defined as follows:

$${d}_{i}={\gamma }_{0}+{\gamma }_{1}{X}_{1i}+{\gamma }_{2}{X}_{2i}+\dots +{\gamma }_{q}{X}_{qi}+{\varphi }_{i}$$

where ${\varphi }_{i}$ is the error term, which assumes zero conditional mean, $E({\varphi }_{i}|{X}_{1i},\dots {X}_{qi})$=0. The coefficients ${\gamma }_{i}$ are the marginal effects of each factor in ${X}_{j}$ on the combined measure of health-income (${d}_{i}$). The regression is optimally estimated using OLS. The sign and the significant level of the coefficients reflect the effect that the variables ${(X}_{j})$ have on the individual components of the index [34].

The coefficients cannot be interpreted as the contribution of each variable to the observed level of inequality; however, we can estimate the importance of the independent variable in the observed inequalities using the logworth values for the regression. The logworth statistic is defined as $-({\mathrm{log}}_{10})$ (p-value of the F test). These values are logarithmic transformations of the p-values from the F test to get the ranking of the variable’s importance [34]. Due to the heterogeneity of the sample, this estimation was done country by country [33, 37].

## Results

### Sample characteristics

The final dataset included only the participants with complete information on the health outcomes and income variables, in total 11,529 participants. Almost half (46%) of the sample were from four countries: China, Australia, Germany, and Switzerland. On average, the participants were 51 years old. South Africa, Morocco, and Romania had a much younger sample, around 38 years old. In general, there were more males with SCI than females (3:1), which is representative of the global incidence of SCI. In Japan and Poland, the number of females with SCI was less than 20%, and in Lithuania and the United States, it was around 40%. There were fewer individuals with tetraplegia, especially in Indonesia, where 88% of the sample had paraplegia. Most causes of SCI were due to trauma (80%). In the United States, we observed only participants with traumatic SCI. Traffic accidents were the most common cause of traumatic SCI across countries.

In terms of the analyzed health outcomes, Switzerland and Japan reported the highest values for the years living with the injury, on average 22 years. In contrast, people with SCI in China and Brazil had lived, on average, only 6 and 4.8 years, respectively, with the injury. As for the comorbidity index, rescaled from 0 (more comorbidities) to 100 (fewer comorbidities), people in South Korea reported the smallest value, with 47.5, and the highest value in Brazil, with an indicator of 69.08.

Around 18% of those surveyed reported unmet healthcare needs across countries. In Norway, unmet healthcare needs were reported by 9% of the respondents; in Morocco, it affected 62% of the respondents. Participants with more income (in purchasing power parity-PPP) were concentrated in the United States (USD 52,361), Switzerland (USD 37,218), Australia (USD 33,465), and lower-income in Indonesia (USD 614) (see Table 1).

**Table 1:** Demographic and descriptive information of participants across different countries

	**Age**	**Comorbidity index**	**Years after injury**	**Income**	**Sex = Female**	**Paraplegia**	**Incomplete**	**Unmeet health care needs**	**Non-traumatic SCI**	**Traumatic SCI**			**N**
**Country**										**Work**	**Traffic accidents**	**Falls**	
Australia	56.77	60.45	18.66	33′253.42	26.2%	58.6%	66.6%	16.6%	16.4%	12.9%	33.4%	17.1%	1376
	(58.00)	(60.78)	(14.50)	(24′215.38)									
Brazil	43.94	69.08	4.79	7′480.59	20.6%	59.8%	78.9%	23.6%	29.6%	10.1%	14.1%	17.1%	199
	(42.00)	(71.05)	(3.50)	(4′474.78)									
China	49.67	74.89	6.04	2′498.60	28.9%	66.9%	74.5%	24.1%	32.3%	12.0%	19.9%	23.1%	1354
	(50.00)	(79.63)	(5.50)	(1′542.69)									
France	51.40	56.86	19.45	14′304.54	27.4%	65.8%	58.0%	9.9%	18.8%	10.7%	44.1%	17.5%	383
	(52.00)	(57.89)	(16.50)	(13′025.18)									
Germany	56.60	60.98	15.46	13′556.26	27.7%	48.4%	65.7%	13.3%	20.7%	10.6%	29.0%	23.7%	1418
	(57.50)	(62.96)	(11.50)	(12′129.38)									
Greece	46.87	60.29	16.64	6′195.72	27.4%	67.5%	53.3%	11.2%	14.2%	12.2%	47.7%	14.7%	197
	(47.00)	(62.50)	(15.00)	(3′930.84)									
Indonesia	43.88	70.15	12.58	615.32	32.3%	87.2%	57.9%	12.8%	11.8%	16.9%	15.9%	22.6%	195
	(44.00)	(72.55)	(13.50)	(261.65)									
Italy	50.45	54.01	12.91	11′577.81	26.3%	73.7%	62.1%	12.1%	29.8%	9.1%	41.4%	17.7%	198
	(50.00)	(55.10)	(10.50)	(9′603.68)									
Japan	55.12	61.79	21.60	15′869.40	16.1%	51.3%	35.8%	13.3%	10.0%	31.9%	42.3%	32.3%	279
	(55.00)	(64.00)	(18.50)	(12′534.05)									
Lithuania	43.22	60.29	18.15	3′707.62	36.6%	69.9%	25.0%	13.0%	6.0%	3.7%	41.2%	18.5%	216
	(43.00)	(62.96)	(18.50)	(2′723.99)									
Malaysia	40.23	66.19	11.09	2′344.63	20.4%	67.5%	59.5%	21.5%	13.8%	16.6%	50.9%	14.5%	289
	(38.00)	(68.00)	(7.50)	(1′578.64)									
Morocco	38.68	57.14	8.56	2′497.62	27.5%	74.0%	55.3%	62.3%	22.6%	20.0%	34.5%	21.3%	385
	(37.00)	(57.78)	(5.50)	(1′633.00)									
Netherlands	58.31	66.39	17.60	17′314.42	29.9%	63.6%	69.7%	11.7%	36.8%	10.4%	21.2%	12.1%	231
	(59.00)	(69.57)	(12.50)	(15′428.46)									
Norway	57.68	64.49	10.65	24′326.57	31.2%	56.6%	79.1%	9.1%	29.8%	10.3%	16.6%	28.3%	580
	(61.00)	(64.58)	(9.50)	(20′712.20)									
Poland	46.84	59.05	15.76	3′808.35	16.8%	52.7%	54.0%	25.5%	10.7%	16.6%	31.2%	28.2%	920
	(45.00)	(59.62)	(12.50)	(2′872.50)									
Romania	38.93	57.86	10.51	2′762.28	26.8%	68.5%	66.7%	12.7%	16.4%	12.7%	28.2%	30.0%	213
	(37.00)	(59.52)	(7.50)	(2′420.55)									
South Africa	38.54	62.41	12.01	3′699.84	24.4%	58.9%	46.7%	27.4%	7.6%	4.1%	31.5%	7.1%	197
	(37.00)	(65.91)	(9.00)	(1′688.25)									
South Korea	48.86	47.58	18.03	11′809.08	24.2%	58.8%	42.1%	27.5%	8.0%	14.6%	47.2%	21.7%	851
	(49.00)	(48.15)	(17.50)	(7′623.33)									
Spain	50.41	62.81	17.46	10′464.56	29.8%	60.9%	52.7%	6.7%	21.3%	14.4%	38.6%	14.7%	389
	(50.00)	(64.58)	(14.50)	(8′914.72)									
Switzerland	58.09	64.22	22.66	37′316.68	29.1%	69.6%	55.5%	7.5%	19.3%	12.7%	28.5%	22.2%	1163
	(58.00)	(65.96)	(20.50)	(33′353.38)									
Thailand	45.81	70.39	10.53	2′640.97	28.6%	73.6%	54.7%	10.0%	13.8%	11.6%	50.8%	15.1%	311
	(44.00)	(74.00)	(7.50)	(1′616.45)									
USA	44.80	62.35	14.00	52′144.32	41.6%	62.2%	70.8%	11.9%	0.0%	2.2%	31.9%	11.4%	185
	(43.00)	(64.44)	(10.50)	(48′384.88)									
Total	51.21	62.36	14.91	15′330.73	26.8%	61.6%	60.5%	17.9%	19.0%	12.9%	32.0%	21.2%	11,529
	(52.00)	(63.83)	(11.50)	(7′866.93)									

Table 2 shows the types of unmet healthcare needs by country. The cost of healthcare was the most reported barrier. In Morocco, this value was 80%, followed by China 52% and Brazil at 47%. The unavailability of service was over 30% in Italy and Spain. In South Africa and Morocco, transportation problems due to cost and availability were 70% and 66%, respectively. Being badly treated was high in China (34%), Romania (30%), Italy (25%), and Poland (23%). Interestingly, in Japan, 49% of the respondents reported as the main barrier to accessing health care the impossibility to take time off from work. Inadequate providers were reported by over 45% of the respondents in France, the Netherlands, and Norway. Lack of information was relevant in China 30%, followed by Greece 27% and Germany 21%.

**Table 2:** Types of unmet healthcare needs* in each country

Country	Healthcare costs	Unavailable service	Transportation	Badly treated	Work related	Inadequate provider	Lack of information	Other
Australia	31%	19%	13%	12%	9%	26%	12%	41%
Brazil	47%	6%	23%	17%	2%	11%	2%	28%
China	52%	18%	23%	34%	5%	15%	30%	19%
France	8%	5%	8%	0%	5%	55%	11%	16%
Germany	16%	22%	11%	7%	4%	20%	21%	60%
Greece	36%	27%	32%	5%	0%	41%	27%	18%
Indonesia	40%	24%	12%	4%	0%	28%	8%	16%
Italy	38%	33%	21%	25%	0%	17%	4%	13%
Japan	5%	3%	5%	5%	49%	11%	11%	46%
Lithuania	39%	18%	21%	14%	0%	32%	4%	57%
Malaysia	26%	11%	34%	5%	18%	13%	10%	27%
Morocco	80%	16%	66%	12%	0%	12%	13%	10%
Netherlands	4%	19%	11%	11%	4%	48%	19%	52%
Norway	15%	25%	8%	17%	0%	40%	6%	45%
Poland	31%	22%	23%	23%	3%	26%	17%	49%
Romania	15%	26%	26%	30%	7%	22%	7%	33%
South Africa	24%	4%	70%	6%	2%	7%	2%	9%
South Korea	22%	20%	19%	10%	13%	20%	13%	24%
Spain	12%	31%	4%	4%	8%	8%	19%	65%
Switzerland	27%	16%	8%	9%	4%	22%	14%	40%
Thailand	29%	13%	39%	10%	3%	23%	0%	23%
USA	23%	14%	9%	5%	9%	32%	9%	73%
Total	36%	18%	25%	16%	6%	21%	16%	32%

Notes: *One participant could choose more than one option

### Years living with the injury

#### Concentration index

**Fig. 1:**
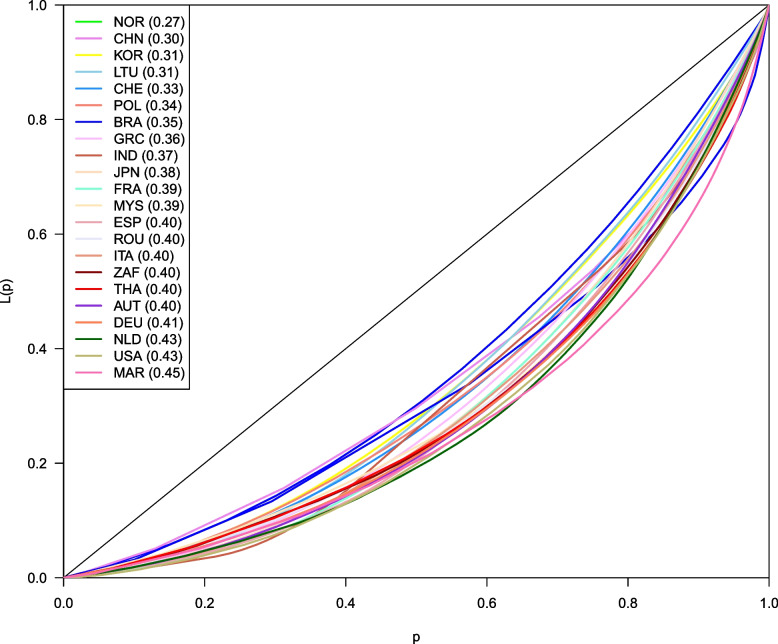
Estimated Lorenz curve for the health outcome: years living with the injury

Figure 1 displays the Lorenz curve for the first health outcome, years living with the injury. All countries showed unequal distributions, as the curves were far from perfect equality (45 degrees). Morocco had the most unequal distribution, with a Gini index of 0.45 (pink line), followed by the USA and the Netherlands with a Gini index of 0.43. On the other extreme (more equal situation), we found Norway with a Gini index of 0.27 (green line), closely followed by China with 0.30. In the middle, we see Japan, France, and Malaysia with values of around 0.38.

Table 3 displays the Erreygers concentration index. A significant concentration of health outcomes in terms of income was shown in nine countries: China, Switzerland, Poland, Germany, Malaysia, Lithuania, Japan, Greece, and South Africa. Only China showed a pro-poor situation (negative coefficient: –0.01), where better health was more concentrated among the lower-income participants. In other countries, the coefficients varied from 0.03 in Switzerland to 0.07 in South Africa, confirming that better health (more years living with the injury) was heavily concentrated among participants in high-income positions.

**Table 3:** Concentration index for years living with the injury

Country	Coef	Standard Error	*p*-value
Morocco	-0.02	0.01	0.17
Brazil	-0.01	0.01	0.12
Italy	-0.01	0.02	0.57
China^a^	-0.01	0.00	0.03
South Korea	0.00	0.01	0.66
Australia	0.00	0.01	0.81
Indonesia	0.00	0.02	0.94
Norway	0.01	0.01	0.14
Thailand	0.01	0.01	0.30
Romania	0.02	0.01	0.18
Netherlands	0.02	0.02	0.35
France	0.02	0.02	0.20
Spain	0.02	0.02	0.14
Switzerland^a^	0.03	0.01	0.00
Poland^a^	0.03	0.01	0.00
Germany^a^	0.04	0.01	0.00
USA	0.04	0.02	0.09
Malaysia^a^	0.04	0.01	0.01
Lithuania^a^	0.04	0.02	0.02
Japan^a^	0.05	0.02	0.02
Greece^a^	0.06	0.02	0.00
South Africa^a^	0.07	0.02	0.00

Notes: If the concentration index is positive means that years living with the injury are concentrated among individuals with a higher income position, if it is negative the concentration of the health outcome is among the lowest income position individuals

^a^The index is statistically significant different from zero (*p* value < 0.05)

### ***Decomposition of the inequality***

Considering the part of the inequality that can be explained by the selected factors, age at the time of the injury appeared as the main factor explaining health–income inequalities in 19 of 22 countries. However, the size of the effect varied considerably by country, ranging from almost 92% in Switzerland to 2% in Brazil.

**Fig. 2:**
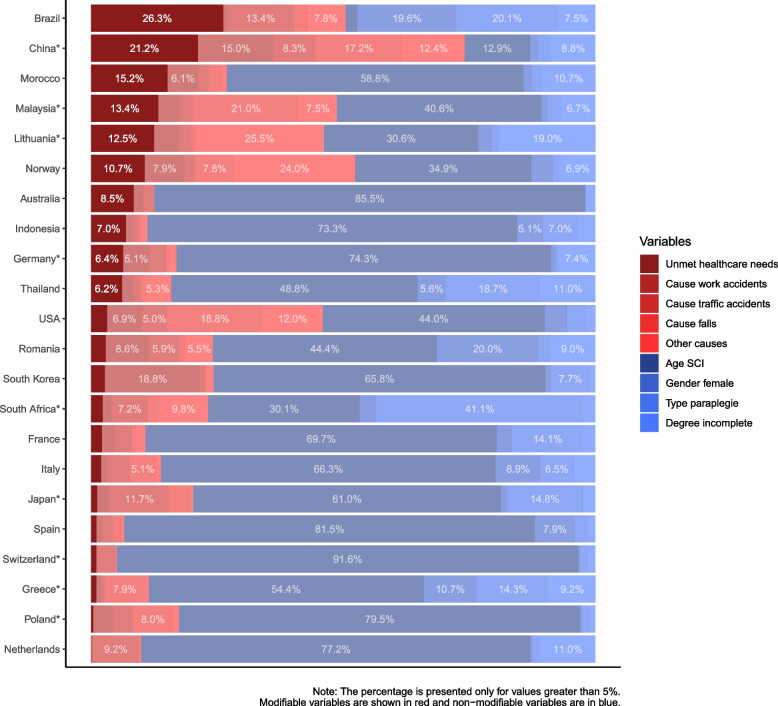
Decomposition of inequality for the health outcome: years living with the injury

Other important factors explaining inequality were *unmet healthcare needs* and the *cause of the injury*. Considering only countries with a significant concentration index, *unmet healthcare needs* explained 21% of the observed inequality in China and 13% in Lithuania and Malaysia. *Unmet healthcare needs* provide no or very low explanation of inequality in Poland, Greece, and South Africa. The type of injury was an important factor in South Africa (41%), while falls explained 21% of the inequality in Malaysia and 17% in China (see Fig. 2). Table A2 in the appendix shows the coefficients for each factor and country as a result of the direct regression.

### Comorbidity index 

#### Concentration index

**Fig. 3:**
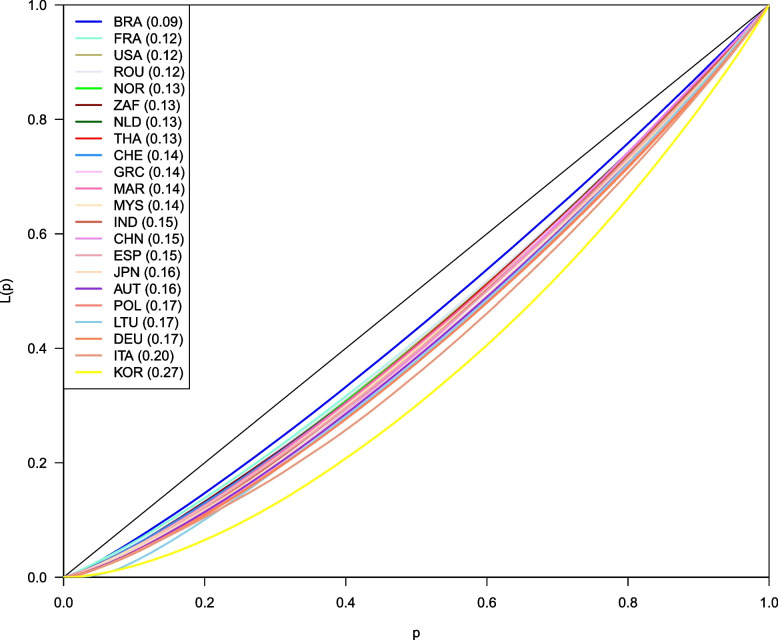
Estimated Lorenz curve for the health outcome: comorbidity index

An unequal distribution was also observed in terms of the comorbidity index but to a smaller extent. The Gini index ranged from 0.09 (less unequal) in Brazil to 0.27 (more unequal) in South Korea. In other countries, the concentration index ranged between 0.17 and 0.12, with the exception of Italy where the Gini index was 0.20 (see Fig. 3).

The concentration of comorbidities across income distribution was significant in Italy, Japan, Spain, Norway, Poland, South Korea, Germany, Australia, Romania, and China. In Italy and Japan, the coefficients were slightly negative, showing a pro-poor situation: better health status or fewer comorbidities among the poor. The rest of the countries showed a pro-rich situation (better health in richer groups). The highest pro-rich inequality was in China (0.15) (see Table 4).

**Table 4:** Decomposition index for Comorbidities

Country	Coef	Standard Error	*p*-value
Italy*	-0.11	0.04	0.00
Japan*	-0.06	0.03	0.02
France	-0.03	0.02	0.21
Indonesia	-0.02	0.04	0.55
Netherlands	-0.01	0.03	0.82
Morocco	0.00	0.02	0.98
USA	0.00	0.03	0.98
Brazil	0.00	0.03	0.97
Malaysia	0.00	0.03	0.88
Switzerland	0.01	0.01	0.68
Greece	0.01	0.04	0.86
Thailand	0.01	0.02	0.67
Lithuania	0.02	0.03	0.61
Spain*	0.05	0.02	0.04
Norway*	0.05	0.02	0.01
South Africa	0.05	0.03	0.11
Poland*	0.06	0.01	0.00
South Korea*	0.07	0.02	0.00
Germany*	0.07	0.01	0.00
Australia*	0.08	0.01	0.00
Romania*	0.09	0.03	0.00
China*	0.15	0.01	0.00

**Notes:** If the concentration index is positive means that the better health status (less comorbidities) are concentrated among individuals with a higher income position, if it is negative the concentration of the health outcome is among the lowest income position individuals

^*^The index is statistically significant different from zero (*p *value < 0.05)

### ***Decomposition of the inequality***

*Unmet healthcare needs* were the main factor explaining health–income inequality for the comorbidity index. This result varied by country from 62.7% in Morocco to 1% in Indonesia. In 14 of the 22 countries, the importance of *unmet healthcare needs* fell above 20%.

Considering the countries where the concentration of comorbidities was statistically significant due to income, *unmet healthcare needs* showed the highest values in Australia (61%) and Germany (40%). China, Norway, South Korea, and Japan showed values between 20 and 33%. This participation of *unmet healthcare needs* in the observed inequality fell under 10% in Poland, Romania, and Spain. Italy with the lowest participation at 8.7%.

**Fig. 4:**
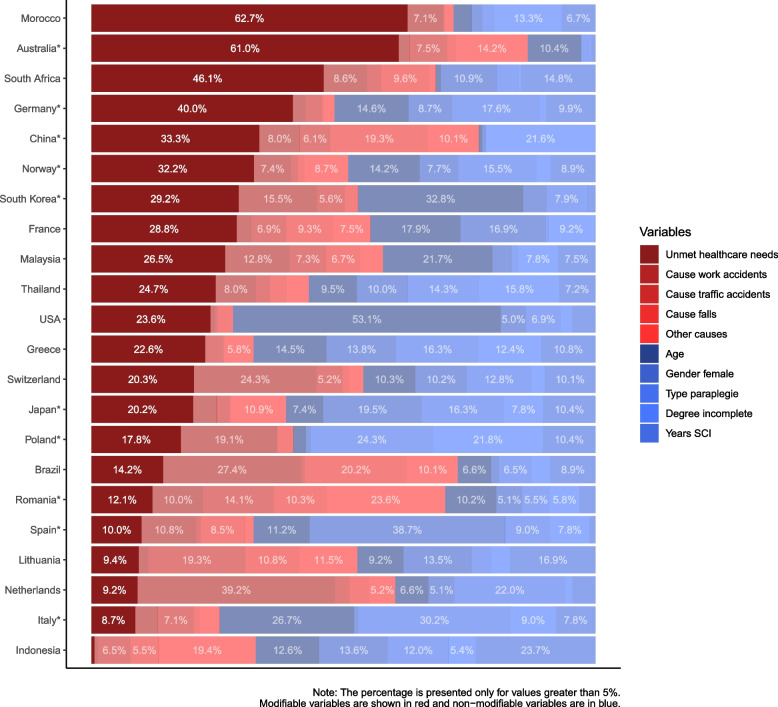
Decomposition of inequality for the health outcome: comorbidity index

The type of injury and the age of participants were also important factors explaining health–income inequality in the comorbidity index. The type of injury was higher in Italy, Poland, and Germany at 30%, 24%, and 18%, respectively, while the age of participants was higher in South Korea (33%) and Italy (27%). The degree of injury was highly relevant in China and Poland, at 22%. Gender was important in Spain (39%) and Japan (20%) (see Fig. 4). The coefficients of each factor and country as a result of the direct regression are displayed in the appendix Table A3.

## Discussion 

These results show that, on average, both analyzed health outcomes —years living with the injury and the comorbidity index—displayed a pro-rich concentration; that is, better health status was concentrated among higher-income groups rather than in low-income groups. The main cause of such inequality is explained by modifiable factors, such as *unmet healthcare needs*, and to a lesser extent by non-modifiable factors, such as age. This result was observed across all countries in the sample, with a more unequal distribution for years living with the injury than for the comorbidity index. Age was the most important factor of inequality in the distribution of years living with injury, followed by the cause of the injury, in which work and traffic accidents showed the highest values. These results highlight the relevance of the living conditions of the participants before the injury because some people have more probability to get an injury. For the comorbidity index, unmet healthcare needs were more important in explaining the observed inequality.

The implemented approach allows us to better understand the reasons behind health inequalities for people with long-term disabilities, such as people living with SCI. Focusing on the groups that most need and use health services, and understanding the differences between countries generate evidence to better design national policies aiming at targeting health inequalities. In addition, our study identifies the part of the inequality that we can change through the health system. Thus, our study expands the current efforts to better understand the problems and challenges of people with SCI around the world, providing evidence of the role of unmet healthcare needs in the range of health inequality in 22 countries.

In spite of the several studies on health inequality and concentration index, there are few studies focusing on people with SCI, and even fewer comparing the situation across countries. The contribution of this paper is that it documents evidence on health inequalities and country experience, which are needed to support the policymakers. The use of the InSCI survey allows us to show different realities worldwide, which implies that standard solutions may not be useful for everyone.

Finally, the direct regression approach to decomposing the socioeconomic inequality of health show how modifiable and non-modifiable determinants of health determine the concentration of health by income level. In addition, this provides a framework for developing deeper and larger health equity studies and extended to other populations.

### Limitations

Our study had limitations. We acknowledge potential selection bias related to the nonrandom collection of data in some countries. Furthermore, the use of self-reported data [19] could generate further bias and an underestimation of the results. However, standard measures were used to allow the comparison between countries, and the analysis was done country by country. The fact that in some countries there was no significant difference between high- and low-income groups does not mean that there is no inequality in those countries. It is important to keep in mind that the data are not representative of the entire SCI population; rather, it provides a description of the situation in each country and generates evidence to be tested in future studies. As mentioned, there are many factors that determine health, and the complexities between interrelated variables make a simple relationship complicated to establish [10]. Nevertheless, this study provides a general approach to the concentration of health and the composition of the inequalities, considering the most important variables that affect the health of people with SCI [24]. While some studies included mortality rates or life expectancy to represent health inequality [42], this paper includes proxy variables that are data-driven to provide information about comorbidities and those who live more years living with the injury. Finally, our findings are descriptive and do not allow us to infer causality and the selected factors do not explain the whole health income inequality.

Generating evidence about the role of the unmet healthcare needs in a population that faces an irreversible, long-term health condition characterized by a high level of limitation and restriction and several comorbidities contributes to the analysis of healthcare systems. Persons with disabilities experience persistent health inequality, which claims an urgent action. From a macro perspective, it is important to put people with disabilities at the center of attention in inequality studies. However, for these purposes, it is important to measure inequalities, identify the factors that determine gaps, and understand the specific realities in each country. In the case of SCI, not everyone is equally likely to suffer an injury, it depends on many country-specific factors, where people in lower-income groups are at disadvantage. This study documents evidence of experiences in different contexts and aims to stimulate action at the country level.

## Conclusion

*Unmet healthcare needs* explain a big part of the socioeconomic inequality of health in people with Spinal Cord Injury, however, this is not a unique factor. This reality is prevalent in low, middle, and high-income countries, with disproportionate effects on people that are highly dependent on the effectiveness of the health system, such as people facing disabilities. In order to achieve health equity, it is important to quantify the size of health inequalities and identify the main driving factors to better design policies aiming at improving the provision of health services to vulnerable populations.

## Acknowlegements

This study is based on data from the International Spinal Cord Injury (InSCI) Community Survey (Ref. Am J Phys Med Rehabil. 2017;96[suppl]: S23-S34). The members of the InSCI Steering Committee are J. Middleton, J. Patrick Engkasan, G. Stucki, M. Brach, J. Bickenbach, C. Fekete, C. Thyrian, L. Battistella, J. Li, B. Perrouin-Verbe, C. Gutenbrunner, C. Rapidi, L.K. Wahyuni, M. Zampolini, E. Saitoh, B.S. Lee, A. Juocevicius, N. Hasnan, A. Hajjioui, M.W.M. Post, J.K. Stanghelle, P. Tederko, D. Popa, C. Joseph, M. Avellanet, M. Baumberger, A. Kovindha, and R. Escorpizo.

## Supplementary Information

**Additional file 1.** Appendix 

## Data Availability

The data that support the findings of this study are available from the International Spinal Cord Injury Community Survey (InSCI) Study Center but restrictions apply to the availability of these data, which were used under license for the current study, and so are not publicly available. Data are however available from the authors upon reasonable request and with permission of InSCI Study Center. To request the data please contact with Ana Oña ana.ona@paraplegie.ch.

## Footnotes

1 This approach uses level-dependent indices and not rank-dependent indices, which are based upon income levels. The basic version of the level-dependent index could be defined as: $L=\frac{1}{n}{\sum }_{i=1}^{n}({\widetilde{y}}_{i}{h}_{i}-{\mu }_{h})$=$\frac{1}{n}{\sum }_{i=1}^{n}{d}_{i}={\mu }_{d}$
