# Supplementary material for: Unmet healthcare needs and health inequalities in people with spinal cord injury: a direct regression inequality decomposition
Source: Int J Equity Health. 2023 Mar 30;22:56. doi: 10.1186/s12939-023-01848-z (PMC10060928; doi:10.1186/s12939-023-01848-z)
Supplement: Supplementary file 1 — Additional file 1. Appendix [file 12939_2023_1848_MOESM1_ESM.docx]

Appendix

A1. Comorbidity index

The comorbidity index (${Com}_{i}$) is the result of the sum of 15 secondary health conditions reported, and weighted by 1) the severity ($T_{ip}$), and 2.) their correlation with mortality ($w_{p}$).

$${Com}_{i}=\sum_{p=1}^{15} w_{p}*T_{ip}$$

The value of 2 was assigned for health problems with a "chronic" severity reported ($T_{ip}$), 1 for "mild or moderate", and 0 for "no problem". For the mortality weights, the values go from 1 for health problems with a low mortality risk to 3 for high mortality risk.

***High mortality risk (3):*** respiratory problems and circulatory problems

***Mild mortality risk (2):*** bowel dysfunctions, urinary tract infections, bladder dysfunction, muscle spasms-spasticity, pressure sores-decubitus, injured caused by loss of sensation, autonomic dysreflexia, depression

***Low mortality risk (1):*** sleeping problems, sexual dysfunction, contractures, postural hypotension, pain

A2. Direct regression results for the composite variables: years after injury and income

|  | **Age SCI** | **Sex= Female** | **Paraplegia** | **Incomplete** | **Unmeet health care needs** | **Work** | **Traffic** | **Falls** | **Other traumatic causes** | **Constant** | **N** |
| --- | --- | --- | --- | --- | --- | --- | --- | --- | --- | --- | --- |
| **Country** |  |  |  |  |  |  |  |  |  |  |  |
| Australia | -0.490*** | -0.160 | 0.0709 | -2.173* | -5.358*** | 2.776* | 2.444* | 0.230 | -0.120 | 20.45*** | 1351 |
|  | (-17.59) | (-0.15) | (0.07) | (-1.99) | (-4.98) | (2.07) | (2.06) | (0.19) | (-0.11) | (10.74) |  |
| Brazil | 0.00487 | 2.436 | -1.256 | -0.801 | -1.174 | 0.107 | 1.840 | 0.238 | 0.717 | 0.163 | 198 |
|  | (0.28) | (1.55) | (-1.57) | (-0.76) | (-1.89) | (0.08) | (1.18) | (0.28) | (0.79) | (0.09) |  |
| China | -0.0825*** | 0.323 | -0.472 | 0.974** | -1.548*** | -2.032*** | -1.447** | -1.680*** | -1.502*** | 4.637** | 1353 |
|  | (-3.44) | (0.87) | (-1.22) | (2.73) | (-4.58) | (-3.76) | (-2.63) | (-4.07) | (-3.36) | (3.08) |  |
| France | -0.361*** | -1.658 | 4.053* | -1.238 | 1.541 | 1.832 | 2.104 | -0.590 | 1.411 | 9.702* | 371 |
|  | (-5.30) | (-0.65) | (2.02) | (-0.54) | (0.50) | (0.57) | (0.73) | (-0.22) | (0.42) | (2.10) |  |
| Germany | -0.388*** | -0.873 | 0.538 | -4.143*** | -4.175*** | 5.487** | 2.982* | -0.754 | -1.301 | 19.58*** | 1382 |
|  | (-13.61) | (-0.93) | (0.59) | (-3.83) | (-3.52) | (3.10) | (2.35) | (-0.81) | (-1.32) | (9.54) |  |
| Greece | -0.389*** | 5.662* | -7.499* | -5.444 | 1.475 | -0.833 | -0.754 | -4.996 | -0.898 | 21.33*** | 192 |
|  | (-5.73) | (2.12) | (-2.55) | (-1.92) | (0.37) | (-0.33) | (-0.25) | (-1.74) | (-0.29) | (3.86) |  |
| Indonesia | -0.494*** | -1.928 | -4.242 | 1.657 | 5.523 | -1.104 | -1.845 | 0.414 | -1.325 | 19.55*** | 179 |
|  | (-6.16) | (-1.11) | (-1.40) | (0.83) | (1.40) | (-0.32) | (-0.40) | (0.11) | (-0.39) | (3.57) |  |
| Italy | -0.246*** | -2.083 | -1.887 | -1.207 | -0.881 | -0.623 | -1.531 | -2.023 | -0.604 | 13.28** | 196 |
|  | (-4.03) | (-1.08) | (-0.85) | (-0.62) | (-0.32) | (-0.19) | (-0.64) | (-0.71) | (-0.17) | (2.82) |  |
| Japan | -0.646*** | -1.498 | 8.344** | 2.749 | 2.388 | 2.524 | -9.368* | -4.621 | 0.950 | 22.96*** | 266 |
|  | (-6.61) | (-0.49) | (2.86) | (0.87) | (0.48) | (0.84) | (-2.47) | (-1.28) | (0.20) | (3.87) |  |
| Lithuania | -0.449*** | -2.198 | 1.243 | 7.282** | -7.581* | 10.46 | -2.366 | 0.736 | -9.777*** | 16.53*** | 215 |
|  | (-4.39) | (-0.88) | (0.54) | (3.30) | (-2.56) | (1.37) | (-0.83) | (0.28) | (-3.95) | (3.57) |  |
| Malaysia | -0.242*** | -0.738 | 0.986 | -2.129 | -3.398 | -2.020 | -1.118 | 7.320** | 4.781 | 9.246** | 275 |
|  | (-3.91) | (-0.30) | (0.62) | (-1.21) | (-1.97) | (-0.88) | (-0.56) | (2.63) | (1.32) | (2.94) |  |
| Morocco | -0.181*** | 0.511 | 0.528 | 1.549 | -2.230* | -1.612 | -0.561 | -0.712 | 0.873 | 5.978** | 385 |
|  | (-4.92) | (0.42) | (0.45) | (1.72) | (-2.18) | (-1.16) | (-0.50) | (-0.58) | (0.28) | (2.62) |  |
| Netherlands | -0.507*** | 0.287 | -1.083 | -5.610 | -0.0961 | -0.319 | 5.958 | -0.428 | 0.000265 | 25.33*** | 225 |
|  | (-6.15) | (0.11) | (-0.40) | (-1.87) | (-0.03) | (-0.08) | (1.65) | (-0.14) | (0.00) | (4.52) |  |
| Norway | -0.0517* | -0.353 | 0.114 | -0.594 | -1.157 | 0.924 | 0.247 | -0.584 | 1.342 | 2.927 | 570 |
|  | (-2.10) | (-0.44) | (0.15) | (-0.65) | (-0.91) | (0.72) | (0.23) | (-0.71) | (1.64) | (1.54) |  |
| Poland | -0.382*** | -0.158 | 1.081 | 0.763 | -0.397 | -2.188 | -2.658 | -3.324** | -1.105 | 14.79*** | 904 |
|  | (-9.85) | (-0.11) | (0.93) | (0.68) | (-0.33) | (-1.67) | (-1.69) | (-2.68) | (-0.71) | (6.32) |  |
| Romania | -0.213*** | 4.309* | -0.865 | -2.301 | -1.267 | -3.909 | -2.706 | -0.572 | 2.675 | 8.858* | 212 |
|  | (-3.82) | (2.34) | (-0.48) | (-1.36) | (-0.58) | (-1.32) | (-1.00) | (-0.28) | (0.94) | (2.39) |  |
| South Africa | -0.293** | -1.620 | -7.842*** | 1.055 | -1.139 | 2.420 | -4.229 | 2.767 | -5.462 | 18.46*** | 188 |
|  | (-3.11) | (-0.65) | (-3.76) | (0.50) | (-0.49) | (0.38) | (-1.20) | (0.47) | (-1.49) | (3.81) |  |
| South Korea | -0.439*** | 0.813 | 2.776** | 0.731 | 1.705 | 8.688*** | -1.170 | 0.303 | -1.620 | 10.85*** | 805 |
|  | (-9.15) | (0.68) | (2.69) | (0.68) | (1.38) | (4.57) | (-0.65) | (0.17) | (-0.84) | (3.76) |  |
| Spain | -0.419*** | -3.672 | 1.524 | -0.945 | -1.306 | 1.127 | 1.715 | 1.356 | -0.835 | 14.77*** | 382 |
|  | (-7.71) | (-1.92) | (0.89) | (-0.52) | (-0.44) | (0.45) | (0.70) | (0.55) | (-0.33) | (4.00) |  |
| Switzerland | -0.480*** | -0.397 | 1.429 | -1.427 | -2.383 | -0.408 | 3.595 | -0.0515 | 0.381 | 17.61*** | 1095 |
|  | (-12.69) | (-0.31) | (1.11) | (-1.04) | (-0.86) | (-0.19) | (1.96) | (-0.04) | (0.26) | (6.07) |  |
| Thailand | -0.209*** | 1.721 | 3.337* | -2.587 | -2.012 | -1.436 | -0.655 | 0.496 | -2.464 | 7.260* | 303 |
|  | (-4.55) | (1.13) | (2.55) | (-1.81) | (-1.22) | (-0.56) | (-0.36) | (0.21) | (-1.09) | (2.39) |  |
| USA | -0.300*** | -1.936 | 1.824 | 0.905 | 2.858 | -6.410 | -4.745 | -8.831* | -8.319 | 16.81** | 184 |
|  | (-3.51) | (-0.70) | (0.67) | (0.32) | (0.56) | (-1.02) | (-0.80) | (-2.06) | (-1.52) | (2.64) |  |

A3. Direct regression results for the composite variables: comorbidity index and income

|  | **Age** |  | **Sex= Female** | **Paraplegia** | **Incomplete** | **Unmeet health care needs** | **Work** | **Traffic** | **Falls** | **Other traumatic causes** | **Constant** | **N** |
| --- | --- | --- | --- | --- | --- | --- | --- | --- | --- | --- | --- | --- |
| **Country** |  | **Years SCI** |  |  |  |  |  |  |  |  |  |  |
| Australia | -0.233** | -0.0378 | 0.467 | 1.739 | -0.380 | -20.47*** | 3.206 | 6.594* | -2.509 | 9.612** | 13.16* | 1351 |
|  | (-2.66) | (-0.42) | (0.17) | (0.69) | (-0.14) | (-7.29) | (0.90) | (2.15) | (-0.77) | (3.21) | (2.11) |  |
| Brazil | 0.158 | -0.857 | 2.120 | 4.771 | 4.022 | -10.06 | -22.55* | -1.173 | -18.14 | -10.34 | 1.828 | 198 |
|  | (0.77) | (-0.97) | (0.23) | (0.76) | (0.48) | (-1.37) | (-2.14) | (-0.11) | (-1.75) | (-1.07) | (0.12) |  |
| China | 0.101 | -0.0270 | 2.606 | 0.677 | 25.60*** | -30.49*** | -20.47*** | -15.95*** | -26.11*** | -20.49*** | 1.155 | 1353 |
|  | (1.07) | (-0.09) | (0.93) | (0.25) | (8.45) | (-10.67) | (-4.90) | (-4.20) | (-7.95) | (-5.58) | (0.17) |  |
| France | 0.332* | 0.00718 | -9.716* | -0.444 | 6.363 | -14.64** | -3.797 | 6.308 | -7.034 | 7.436 | -19.59* | 371 |
|  | (2.15) | (0.04) | (-2.08) | (-0.10) | (1.38) | (-2.91) | (-0.57) | (1.12) | (-1.38) | (1.19) | (-2.02) |  |
| Germany | 0.224** | 0.209* | -4.907 | 7.113** | 1.005 | -15.57*** | 3.406 | 2.915 | 0.451 | 1.922 | -16.70** | 1382 |
|  | (2.69) | (2.10) | (-1.93) | (3.01) | (0.38) | (-4.85) | (0.78) | (0.98) | (0.15) | (0.68) | (-2.78) |  |
| Greece | -0.361 | 0.331 | 11.15 | 10.80 | 8.398 | -17.05 | 0.276 | 3.348 | 0.275 | 6.105 | -4.200 | 192 |
|  | (-1.40) | (1.13) | (1.35) | (1.52) | (1.25) | (-1.90) | (0.03) | (0.45) | (0.03) | (0.70) | (-0.28) |  |
| Indonesia | -0.349 | 0.747 | 9.700 | -13.72 | 4.868 | -0.959 | 1.220 | 10.17 | -7.684 | -18.68 | 17.88 | 179 |
|  | (-1.19) | (1.86) | (1.26) | (-1.15) | (0.62) | (-0.09) | (0.10) | (0.72) | (-0.63) | (-1.62) | (0.93) |  |
| Italy | 0.418* | 0.276 | 0.789 | 12.80* | 5.704 | -7.603 | -3.998 | 5.707 | 1.370 | -4.215 | -41.42** | 196 |
|  | (2.23) | (0.95) | (0.13) | (2.41) | (1.05) | (-1.02) | (-0.60) | (0.88) | (0.19) | (-0.54) | (-3.04) |  |
| Japan | -0.268 | 0.315 | -14.95* | 10.37* | 7.184 | -14.91* | -5.108 | -4.015 | -0.518 | 13.88 | 4.434 | 266 |
|  | (-1.20) | (1.52) | (-2.32) | (2.07) | (1.24) | (-2.37) | (-0.85) | (-0.53) | (-0.07) | (1.57) | (0.33) |  |
| Lithuania | -0.511 | 0.910* | -14.60* | 5.917 | 4.843 | -12.75 | -8.038 | -18.58** | 15.44 | -13.98* | 18.55 | 215 |
|  | (-1.70) | (2.53) | (-2.20) | (0.90) | (0.89) | (-1.72) | (-0.51) | (-2.75) | (1.89) | (-1.97) | (1.37) |  |
| Malaysia | 0.387 | 0.256 | 3.270 | 0.975 | 4.291 | -12.00 | -10.60 | -5.718 | -5.816 | 5.877 | -14.59 | 275 |
|  | (1.66) | (0.76) | (0.44) | (0.17) | (0.78) | (-1.90) | (-1.15) | (-0.74) | (-0.69) | (0.50) | (-1.10) |  |
| Morocco | 0.150 | -0.333 | -2.956 | 3.235 | 9.510* | -25.61*** | -9.324 | 0.482 | -0.177 | -4.550 | 8.310 | 385 |
|  | (0.93) | (-1.50) | (-0.66) | (0.70) | (2.36) | (-5.92) | (-1.55) | (0.09) | (-0.03) | (-0.54) | (0.87) |  |
| Netherlands | 0.196 | 0.136 | -4.616 | 12.48 | 1.655 | -10.87 | -28.66** | -3.685 | 5.437 | -5.265 | -15.74 | 225 |
|  | (0.82) | (0.60) | (-0.67) | (1.95) | (0.23) | (-1.05) | (-2.82) | (-0.42) | (0.54) | (-0.67) | (-0.92) |  |
| Norway | 0.296* | 0.682 | -7.115 | 9.900** | 3.646 | -21.40*** | -10.01 | -2.347 | 1.955 | 8.101 | -29.01** | 570 |
|  | (2.56) | (1.90) | (-1.72) | (2.71) | (0.82) | (-4.18) | (-1.68) | (-0.45) | (0.45) | (1.86) | (-3.07) |  |
| Poland | 0.116 | 0.390** | 1.733 | 13.55*** | 12.12*** | -10.97*** | -14.87*** | -0.354 | -4.042 | 0.0724 | -17.70* | 904 |
|  | (1.00) | (2.65) | (0.46) | (4.37) | (4.11) | (-3.65) | (-3.80) | (-0.09) | (-1.17) | (0.02) | (-2.45) |  |
| Romania | 0.241 | 0.126 | 3.300 | 3.785 | -3.908 | -9.654 | -9.516 | 10.98 | 7.218 | 15.99 | -15.27 | 212 |
|  | (0.90) | (0.35) | (0.52) | (0.55) | (-0.58) | (-1.03) | (-0.89) | (1.16) | (0.91) | (1.67) | (-0.82) |  |
| South Africa | 0.0882 | 0.930* | -15.77 | -6.646 | -0.665 | -29.78*** | 30.54 | 9.560 | 27.09 | -4.840 | -0.795 | 188 |
|  | (0.30) | (2.34) | (-1.92) | (-1.05) | (-0.11) | (-4.70) | (1.63) | (0.72) | (1.76) | (-0.37) | (-0.04) |  |
| South Korea | -0.554*** | 0.0781 | 3.988 | 5.211 | 0.286 | -12.21*** | 13.40** | -6.561 | 3.347 | -0.363 | 26.84*** | 805 |
|  | (-4.34) | (0.52) | (1.26) | (1.81) | (0.10) | (-4.06) | (2.79) | (-1.44) | (0.77) | (-0.07) | (3.39) |  |
| Spain | 0.213 | 0.0325 | -14.12** | 4.644 | -4.276 | -9.718 | -8.007 | 0.845 | -6.286 | 2.002 | -4.325 | 382 |
|  | (1.14) | (0.17) | (-2.63) | (0.97) | (-0.87) | (-1.05) | (-1.11) | (0.14) | (-0.93) | (0.26) | (-0.37) |  |
| Switzerland | 0.194* | 0.191 | -5.553 | 6.467* | 2.031 | -15.33** | -14.65*** | -4.202 | -1.331 | 2.511 | -16.04* | 1095 |
|  | (1.97) | (1.94) | (-1.95) | (2.26) | (0.73) | (-3.01) | (-3.35) | (-1.23) | (-0.39) | (0.78) | (-2.35) |  |
| Thailand | 0.260 | 0.341 | 7.997 | 11.26 | 9.877 | -22.02* | -12.29 | -4.094 | -5.884 | -7.592 | -23.89 | 303 |
|  | (1.24) | (1.01) | (1.29) | (1.65) | (1.77) | (-2.36) | (-1.09) | (-0.47) | (-0.55) | (-0.67) | (-1.58) |  |
| USA | 0.826*** | 0.225 | 5.595 | -7.305 | 3.051 | -21.95** | 3.537 | -2.292 | -8.779 | 1.152 | -36.64 | 184 |
|  | (4.38) | (0.87) | (0.92) | (-1.16) | (0.47) | (-2.67) | (0.21) | (-0.12) | (-0.58) | (0.06) | (-1.70) |  |

**Table A4.** International Spinal Cord Injury (InSCI) Community Survey 2018. Ethics committees or review boards approvals in the 22 InSCI countries

| Country name | Name of ethics committee or institutional board responsible for ethics approval of the InSCI study | Approval number | Approval date | Form of consent |
| --- | --- | --- | --- | --- |
| Australia | Sydney Local Health District Human Research Ethics Committee;  Australian Institute of Health and Welfare Ethics Committee | HREC/16/HAWKE/495;  EO2017/1/341 | June 7 2017;  Jan 4 2018 | D |
| Brazil | Comissão de Ética para Análise de Projetos de Pesquisa do Hospital das Clínicas da Faculdade de Medicina da  Universidade de São Paulo, CAPPesq | 97049118.8.0000.0068 | Nov 8 2018 | A |
| China | Ethics committees of the first affiliated hospital of Nanjing Medical University, Nanjing, Jiangsu Province;  Ethics Committee of Sichuan University, Sichuan Province, Chengdu | No. 2018-SR-004 (Jiangsu)  No. K2017053 (Sichuan) | March 7 2018;  Jan 22 2018 | B, C |
| France | Comité de Protection des Personnes | Ref : 180304 | April 10 2018 | A |
| Germany | Ethic Committee of Hannover Medical School | 7374 | Feb 13 2017 | B |
| Greece | Scientific/Ethical Committee of General Hospital 'G. Gennimatas' Athens | 20257/1.8.2016 | Aug 1 2016 | A, B |
| Indonesia | Health Research Ethics Committee, National Institute of Health Research and Development | LB.02.01/2/KE.342/2017 | 15 Nov 2017 | A |
| Italy | Comitato Etico Interaziendale AOU 'Maggiore della Carità' di Novara | ASL BI, ASL NO, ASL VCO ItaSCI, 1, 25-01-2018 | April 27 2018 | A |
| Japan | Research Ethics Committee of Wakayama Medical University | 2079 | July 12 2017 | A |
| Lithuania | Vilnius Regional Committee for the Ethics of Biomedical Research | 158200-17-907-421 | May 9 2017 | A,B |
| Malaysia | Medical Research and Ethics Committee, Ministry of Health | NMRR-16-2747-28885(IIR) | July 14 2017 | A |
| Morocco | Hospital and University Ethics Committee of Fez | 03/17 | July 20 2017 | A |
| Netherlands | Medical Ethics Board University Medical Center Utrecht | WAG/mb/17/024763 | Aug 16 2017 | A |
| Norway | Regional Committee for Medical and Health Research Ethics, South East | 2016/1184/REK sør-øst | Sept 21 2016 | A, B |
| Poland | Bioethical Committee of the Medical University of Lodz | RNN/198/16/KE | July 12 2016 | A, B |
| Romania | Ethical Committee of Rehabilitation Hospital Felix Spa | 2228/06.03.2017 | March 3 2017 | A, B |
| South Korea | Institutional Review Board of National Rehabilitation Center | NRC-2016-05-039 | Nov 9 2016 | A, B |
| South Africa | Biomedical Science Research Ethics Committee of the University of the Western Cape | BM/16/3/24 | Oct 24 2016 | A, C (recorded) |
| Spain | Ethical Committee of Hospital Universitari Vall d’Hebron, Hospital Universitario de Cruces, Hospital  Universitario Materno Infantile de Gran Canaria, Hospital Universitario Virgen del Rocio | PR(ATR)285/2016 | Oct 2016 | A |
| Switzerland | Ethical Committee of Northern and Central Switzerland | 11042 PB_2016-02608 | Dec 21 2016 | A,B |
| Thailand | Ethical Committee of Faculty Medicine, Chiang Mai University | REH-2559-04167 | Dec 9 2016 | A |
| United States | University of Vermont Institutional Review Board | CHRBSS:16-574 | Nov 21 2017 | A,E  Nov 21 2017 |

Note: A=written consent; B=questionnaire completion considered as implicit consent; C=oral consent; D=waiver of consent; E=electronic consent
